# Synchrony in auditory 40-Hz gamma oscillations increases in older age and correlates with hearing abilities and cortical GABA levels

**DOI:** 10.1162/imag_a_00035

**Published:** 2023-12-08

**Authors:** Simon Dobri, J. Jean Chen, Bernhard Ross

**Affiliations:** Rotman Research Institute, Baycrest Centre, Toronto, Canada; Department of Medical Biophysics, University of Toronto, Toronto, Canada

**Keywords:** speech-in-noise loss, aging, feature binding, perception, auditory cortex, gamma-aminobutyric acid

## Abstract

Synchronized 40-Hz gamma oscillations in specific sensory and higher-order thalamocortical networks provide a neural mechanism for feature binding. Aging-related changes in gamma oscillations may cause deficits in auditory feature binding, contributing to impaired speech-in-noise perception. Gamma synchrony is controlled through inhibitory mechanisms mediated by the neurotransmitter γ-aminobutyric acid (GABA), which has been shown to decline in aging. This study investigated aging-related changes in gamma oscillations and how they relate to auditory function and cortical GABA levels. Magnetoencephalograms of 40-Hz auditory steady-state responses (ASSRs) were recorded in young and older adults by presenting amplitude-modulated tones in quiet and mixed with concurrent multi-talker babble noise. Responses in the quiet condition had longer latencies and more prominent amplitudes, indicating the 40-Hz ASSRs in noise were dominated by a sensory component and in quiet by a component involved in higher-order processing. The ASSR amplitudes increased in older adults under both stimulus conditions. However, larger ASSR amplitudes were associated with more severe hearing and speech-in-noise loss only in the noise condition. This suggests the aging-related increase in synchrony of sensory gamma oscillations has a detrimental effect on auditory processing. It may cause increased interference between competing sounds in the central auditory system, making it difficult for the aging auditory system to separate speech features from noise and bind them into a distinct perceptual object. Also in older adults, larger amplitudes of the 40-Hz ASSRs in the quiet condition were associated with higher left auditory cortex GABA concentrations measured with magnetic resonance spectroscopy, supporting GABA’s role in internally generated gamma synchrony in aging.

## 1 Introduction

Sensory perception requires segmenting and integrating the component features of real-world objects into mental representations, referred to as perceptual objects. This process is called feature binding (Treisman, 1996). Synchronized neural oscillations provide a mechanism for effective communication between neurons coding different features, allowing the flexible binding of sensory information in anatomically overlapping neural networks (Y. Ding et al., 2017; Eckhorn et al., 1988; Engel & Singer, 2001; von der Malsburg & Schneider, 1986). Binding through synchrony is a particular manifestation of the general concept of neural communication through temporal coherence (Bonnefond et al., 2017; Buzsáki et al., 1983; Fries, 2005, 2015). Oscillating neurons undergo periodic fluctuations in excitability, which define temporal windows for communication (Arnal & Giraud, 2012; Luo & Poeppel, 2012). Populations of neurons coding features of a particular object oscillate synchronously; thus, the sensory information they code is grouped together because they share the same windows for communication. In contrast, neurons coding features of different objects oscillate asynchronously, so the features they code are bound into separate perceptual objects.

Neural oscillations in different frequency bands have been linked to perceptual binding (Ghiani et al., 2021). Recent models of binding involve the coordination between theta (3-6 Hz) and gamma (30-120 Hz) oscillations (VanRullen, 2016). Also, alpha (7-14 Hz) oscillations recorded with electroencephalography (EEG) (Buergers & Noppeney, 2022; Zhang et al., 2019) and magnetoencephalography (MEG) (Hardy et al., 2023) play an active role in binding. Specifically, gamma oscillations with a characteristic frequency of 40 Hz have been suggested as the mechanism underlying perceptual feature binding (S. Ding et al., 2017; Fries, 2009; Tallon-Baudry & Bertrand, 1999; von der Malsburg, 1995; Widmann et al., 2007).

Alterations in the synchrony of gamma oscillations may underlie functional deficits in various conditions, including neuropsychiatric (Herrmann & Demiralp, 2005; Uhlhaas & Singer, 2010) and cognitive disorders (Mably & Colgin, 2018). Also, gamma oscillations change over the lifespan. Some studies found declines in the amplitude and synchrony of gamma oscillations in healthy aging (Goossens et al., 2016; Murty et al., 2020; Muthukumaraswamy et al., 2010), and in mild cognitive impairment (MCI) and Alzheimer’s disease (AD) (Koenig et al., 2005). Other studies found effects in the opposite direction, that is, increased gamma amplitudes in healthy aging (Christov & Dushanova, 2016; Roland et al., 2011) and AD and MCI patients (Osipova et al., 2006; van Deursen et al., 2011). The findings may vary between these studies because they used different stimuli, looked at gamma oscillations with different frequencies, and were conducted for various sensory modalities. Also, multiple gamma oscillations have distinct functional significance (Buzsáki & Wang, 2012; Castelhano et al., 2014). Aging may impact individual aspects of gamma oscillations differently. Therefore, further research is necessary to uncover the mechanistic link between gamma synchrony and functional changes in aging, which will aid the understanding of brain diseases, injury, and degeneration. The current study investigated the relationship between gamma oscillations and auditory binding deficits in aging.

A reduced ability to understand speech in the presence of background noise, termed speech-in-noise (SIN) loss, is a severe concern for older adults (CHABA, 1988). Though aging-related hearing loss is the primary factor contributing to SIN loss, changes in central auditory processing also play a significant role (Akeroyd, 2008; Frisina & Frisina, 1997; Humes, 2021). Successful SIN understanding requires binding the attended speech and background noise into distinct perceptual objects (Bregman, 2015; Ciocca & Bregman, 1989). A recent study showed a correlation between SIN understanding and figure-background perception of non-speech sounds (Holmes & Griffiths, 2019), indicating that aging-related deficits in auditory feature binding may contribute to SIN loss. The binding-through-synchrony hypothesis suggests aging-related changes in gamma synchrony may underlie these auditory binding deficits.

Multiple studies have investigated the role of gamma oscillations in speech perception (e.g., Giraud & Poeppel, 2012; Ou & Law, 2018; Palva et al., 2002; Zion Golumbic et al., 2013), but only a few addressed the association between changes in gamma oscillations and SIN loss. These studies found associations between increasing SIN loss and reduced resting-state gamma power (Houweling et al., 2020), increased amplitudes of auditory-evoked gamma responses (Ross, Dobri, et al., 2020), and reduced dynamic range of auditory steady-state responses (ASSRs) (Ross & Fujioka, 2016). For this study, we employed the 40-Hz ASSR entrained by a sinusoidally amplitude modulated (AM) tone to investigate how aging-related changes in multiple types of gamma oscillations relate to SIN loss.

ASSR amplitudes are maximal for frequencies around 40 Hz (Hari et al., 1989; Picton et al., 1987; Ross et al., 2000; Vierling-Claassen et al., 2010). The 40-Hz ASSR was previously interpreted as a superposition of multiple evoked middle-latency responses (Galambos et al., 1981; Hari et al., 1989), thus reflecting early sensory processing (Näätänen & Picton, 1987). However, subsequent studies showed several functional characteristics differentiate the 40-Hz ASSR from the auditory-evoked responses, such as the effects of contralateral noise (Kawase et al., 2012) and peripheral and central masking (Galambos & Makeig, 1992a, 1992b). These studies support the interpretation that the 40-Hz ASSR reflects gamma oscillations entrained to the stimulus rhythm, which are more relevant for speech perception than evoked activity (Mahmud et al., 2021). Moreover, the 40-Hz ASSR contains multiple types of gamma oscillations, which can be distinguished by analyzing their amplitude and phase (Ross & Fujioka, 2016).

Gamma oscillations in the 40-Hz range are generated in reciprocally connected thalamocortical circuits consisting of excitatory pyramidal cells and inhibitory interneurons (Llinas et al., 2002; Whittington et al., 1997). The interneurons control synchrony in these networks by releasing the inhibitory neurotransmitter γ-aminobutyric acid (GABA) to periodically inhibit the pyramidal cells (Steriade et al., 1998). Network synchrony crucially depends on the kinetics of GABA-mediated inhibition (X.J. Wang & Buzsáki, 1996) and can be affected by changes in the GABA system (Faulkner et al., 1998; Juhász et al., 1994). A recent meta-analysis found cortical GABA levels decline in aging (Porges et al., 2021). Previously, we showed an aging-related decline in auditory cortical GABA levels was associated with increased SIN loss (Dobri & Ross, 2021). The decrease in GABA levels may affect the synchrony of 40-Hz gamma oscillations underlying feature binding, thus explaining the relationship between GABA and SIN loss.

Several studies have shown a positive relationship between GABA levels and gamma oscillations, such as movement-related gamma synchrony (Gaetz et al., 2011) and gamma power in the sound-induced flash illusion (Balz et al., 2016). Also, administering GABA agonists increased the gamma response to a visual stimulus (Saxena et al., 2021), gamma power during a visuospatial working memory task (Lozano-Soldevilla et al., 2014), and overall resting-state gamma power (Hall et al., 2010). However, other studies showed a negative relationship between GABA and gamma: the visual cortical GABA_A_ receptor density was negatively correlated with the gamma amplitude in response to a visual stimulus (Kujala et al., 2015), and administering a GABA agonist reduced the amplitude of the transient auditory 40-Hz response (Jääskeläinen et al., 1999). Moreover, the relationship between GABA and gamma may be age-dependent. Cortical GABA levels were positively correlated with the coherence of gamma oscillations in children and adolescents, but the correlation was not evident in young adults (Port et al., 2017). Hitherto, it has not been shown whether there is a correlation between GABA and gamma oscillations in healthy aging, and it is not clear whether such a correlation would be positive or negative. The current study investigated behavior, brain function, and metabolism within the same group of older adults, which may help inform how aging impacts speech perception.

We used MEG to record 40-Hz ASSRs in young and older adults. We used a perturbation paradigm for studying multiple components of the ASSR, where we presented a 40-Hz AM tone in quiet and with concurrent multi-talker babble noise (Ross & Fujioka, 2016). We measured bilateral auditory cortical GABA levels using Mescher-Garwood point resolved spectroscopy (MEGA-PRESS) edited magnetic resonance spectroscopy (MRS). We analyzed age effects as group differences and as linear functions of age in the older group. We used linear modeling to reveal correlations between auditory abilities, age, gamma-frequency ASSRs, and GABA levels.

## 2 Methods

### 2.1 Participants

The study protocol followed the principles of the World Medical Organization Declaration of Helsinki (2013) and was approved by the institutional Research Ethics Board (REB 15-30). We recruited young and older healthy adults through the institutional research database. All participants reported that they spoke English as their primary language, were generally healthy, had no history of neurological or psychiatric disorders, and were not taking any neuroactive drugs. Participants were given verbal and written information about the nature of the study before providing written consent and received an honorarium for completing the study.

We previously reported an analysis of the MRS and behavioral data from the same participants in this study (Dobri & Ross, 2021). Twenty-two young and 25 older adults completed the MEG recordings. However, due to time constraints, complete MRS scans were obtained only in 19 young (19-28 years old, mean age 23.8, SD 3.0 years, 11 or 58% female) and 19 older (69-87 years old, mean age 76.1, SD 6.2 years, 12 or 63% female) participants. In this study, we report the data obtained from the 19 young and 19 older participants who completed MEG and MRS sessions. In one case, MEG and MRS were acquired on the same day; otherwise, the sessions were separated by 7.6 days in the mean (maximum of 30 days). The MEG recordings were acquired before the MRS to avoid MEG artifacts caused by residual magnetization from the MR scanner.

### 2.2 Behavioral tests

Hearing thresholds were assessed in all participants with pure tone audiometry using the modified Hughson-Westlake procedure at octave frequencies between 250 Hz and 8,000 Hz for both ears. The four-frequency pure-tone-average (PTA) was calculated as the mean hearing threshold between 500 Hz and 4,000 Hz. These frequencies are most relevant for speech perception in noise (Humes, 2021; Smoorenburg, 1992).

To assess word recognition in noise, we administered the QuickSIN test (Killion et al., 2004) to the older group only, as we would not expect much variation in performance in the young group (Ross et al., 2021). We calculated the signal-to-noise ratio for 50% word recognition (SNR_50_) by approximating a logistic curve to the psychometric function of the QuickSIN scores using the Palamedes Matlab toolbox (Kingdom & Prins, 2016). We reported the difference between SNR_50_ and the normative value of QuickSIN (-2dB) as the individual SIN loss scores.

Both hearing tests were performed in a soundproof booth using a clinical audiometer (GSI61, Grason Stadler, Eden Prairie, MN) and ER-3A sound transducers (Etymotic Research, Elk Grove Village, IL) connected to the participants’ ears with 20 cm of flexible plastic tubing and foam earpieces.

### 2.3 Auditory stimuli

The stimulus for eliciting the ASSR was a 400-Hz tone, sinusoidally amplitude modulated (AM) between 0 and 100% at the modulation frequency of 40 Hz. The stimulus consisted of five short bursts of the AM sound of 400 ms duration, separated by 12.5 ms silent gaps. The gap length was equivalent to one-half period of the 40 Hz AM. The total length of the AM sound was 2,050 ms, and the inter-onset interval was 4,000 ms. Using AM stimulus bursts separated by short gaps instead of a continuous AM sound allowed for observing the temporal dynamics of the brain responses.

Participants listened to the AM stimulus under two experimental conditions. The stimulus was presented alone (the “quiet” condition) or with concurrent multi-talker babble noise consisting of four female French speakers (the “noise” condition). The babble noise was presented in bursts beginning 500 ms before the onset of the AM sound and ending 500 ms after the offset of the AM sound for a total duration of 3,050 ms. The babble noise faded in and out with 200 ms cosine-shaped slopes.

In previous studies, we presented the AM stimulus and the noise to opposite ears to create a central interference condition (Ross & Fujioka, 2016; Ross et al., 2012). For the current study, we separated the AM stimulus and the noise into different spectral bands. The AM sound consisted of three spectral components at the 400-Hz carrier frequency and sidebands at 360 Hz and 440 Hz. Therefore, the noise signal was notch-filtered in the octave around the AM sound (-6 dB at 283 Hz and 566 Hz, <-60 dB at 400 Hz). The notch filter reduced the spectral overlap between the AM sound and the concurrent babble noise, thus minimizing masking at the cochlear level. Because the babble noise and the AM sound did not spectrally overlap, we assumed that interactions occurred at a central stage of auditory processing beyond the tonotopically organized early sensory processing. Therefore, masking of the AM sound by the babble noise was informational rather than energetic (Brungart, 2001; Schneider et al., 2007; Shim & Berg, 2013). The total SNR was 6 dB, calculated as the power of the AM sound divided by the power of the babble noise.

Participants passively listened to the stimuli presented in blocks of 300 s duration, containing 75 trials each, either with or without the concurrent noise. Four blocks in the “quiet” and four in the “noise” condition occurred in alternating order. The stimuli were presented through ER-3A sound transducers connected by 2 m plastic tubing to foam inserts in the participants’ ears. Identical AM stimuli and noise were presented simultaneously to both ears (i.e., diotic stimulation) at an intensity of 60 dB above the individual sensation threshold for the stimulus tone frequency (400 Hz), measured before the MEG recording. The stimulation timing was controlled by Presentation software (Neurobehavioural Systems, Berkeley, CA).

### 2.4 MEG data acquisition and processing

MEG data were recorded in a silent, magnetically shielded room at the Rotman Research Institute in Toronto using a 151-channel whole-head MEG system (CTF-MEG, Port Coquitlam, BC, Canada) with first-order axial gradiometer detection coils (Vrba & Robinson, 2001). At the time of recording, one channel was disabled for technical reasons. Magnetic field data were low-pass filtered at 300 Hz, sampled at 1,250 Hz, and stored continuously. Participants were seated comfortably in an upright position, with their heads resting inside the helmet-shaped MEG device. Participants were instructed to keep their eyes open and blink normally, maintain visual focus on a fixation cross attached to the wall directly in front of the MEG seat, remain as still as possible, and listen passively to the auditory stimuli during recording. The head position was registered at the beginning and end of each block using three detection coils, one attached to the nasion and the others to the left and right pre-auricular points. A data block was rejected if the difference in head position pre- and post-recording was larger than ±4 mm in any direction, ensuring that participant motion had a minimal effect on source localization.

Eye blink and cardiac artifacts were removed without rejecting trials. An independent component analysis (ICA) was applied to a subset of 20 frontal MEG sensors, and the 2 components representing the temporal patterns of eye-blink and eye-movement artifacts were identified. Subsequently, a regression of the 6-Hz low-pass filtered artifact waveforms was subtracted from the MEG data. For detecting cardiac artifacts, we utilized the quasi-periodic nature of the heart beats. After applying an ICA to the full set of MEG data, a cepstrum analysis identified the component with the strongest periodicity in the log-transformed spectrum. Peaks in the ICA waveform indicated the heartbeats. Averaging the MEG according to the peaks provided a cardiac artifact model, which was regressed out from the MEG at the time points of all heart beats.

### 2.5 Source analysis

Cortical source activity of the gamma-band ASSR can be reliably analyzed assuming single dominant sources in primary auditory cortices (Edgar et al., 2016; Herdman et al., 2003; Ross et al., 2002; Tan et al., 2015), even though it likely has multiple functionally distinct neural generators (Pastor et al., 2002). Therefore, we modeled bilateral auditory cortex activity with equivalent current dipoles based on the 40-Hz activity elicited by the AM sound. The MEG trials in each block were parsed into epochs of 500 ms duration beginning at the onset of the stimulus sequence and the onset after the four 12.5 ms stimulus gaps. Ten sub-averages were constructed from all combinations of three out of the five epoch types. With four repeated blocks, 40 sub-averages were available for repeated source analysis for each participant and condition. Single dipoles located in the left and right auditory cortices were fitted simultaneously to the 150-channel magnetic field distribution of the average response using the spatiotemporal fit as implemented in the *dfit* function of the CTF software. The initial model was a pair of mirror symmetric dipoles positioned in the left and right temporal regions. Individual models were constructed iteratively by fitting the dipole in one hemisphere while keeping the other constant, alternating between hemispheres. Source models for each participant were calculated as the mean across the dipole estimates for the sub-averages. Dipole estimates were accepted for the final model if the dipole fit explained at least 90% of the variance in the measured magnetic field and the distance from the mean was less than two times the standard deviation. On average, dipole estimates were successful in 34.7 out of 40 cases (std. dev. 3.6, minimum 25) in the quiet condition. Dipole estimation was more variable in the noise condition, yielding in mean 32.4 successful fits per participant (std. dev. 7.0, minimum 11 in the young and 17 in the older group).

Source space projection of the dipole model was used to construct two source waveforms for each trial (Ross et al., 2000; Teale et al., 2013; Tesche et al., 1995). Source waveforms were estimated after the MEG data were parsed into trials beginning 500 ms before stimulus onset and ending 2,500 ms after stimulus onset, resulting in left and right waveforms of cortical signal strength measured in nano-Ampere metres (nAm). Individual participant data were averaged across all trials, and the DC offset was determined from the mean of the 500 ms pre-stimulus interval and subtracted from the data. The averaged source waveforms were filtered with a one-octave wide FIR bandpass (length of 512 samples, -3-dB frequencies of 28 Hz and 56 Hz). The 40-Hz time series were expanded into the complex domain using the Hilbert transform.

We calculated the ASSR as the discrete Fourier transform at the AM frequency *f_m_* = 40 Hz using the *N* samples of the signal *x(t_n_)*

$$ASSR=\frac{1}{N-1}\sum_{n=1}^{N}x\left(t_{n}\right)\;\cdot \;e^{-i2\pi f_{m}t_{n}}$$

The ASSR is complex-valued; the absolute value is the amplitude of the ASSR, and the phase indicates the phase difference relative to the stimulus. The ASSR was calculated for the N = 188 samples in the 250 ms to 400 ms interval following the stimulus onset and the onsets after the four gaps. The mean of these five values provided the amplitude and phase of the ASSR, which were calculated for each stimulus condition and each participant. The complex amplitudes of the ASSR were obtained as the mean of the cross-spectrum between the complex conjugate waveform of the stimulus AM and the response in the interval spanning 250 ms to 400 ms following the initial onset of the AM tone and after each 12.5 ms gap. The ASSR amplitudes for these five stimulus events were averaged, resulting in four ASSR amplitude measures for each participant: separately for the left and right auditory cortex and for the two stimulus conditions. Significance for the individual ASSR amplitudes was tested with bootstrap randomization. The distribution under the null hypothesis of no phase-locked 40-Hz brain response was estimated by calculating the ASSR amplitude 1,000 times, each time adding a random phase to the data in each trial. The observed ASSR amplitude was compared with the null distribution for all participants and conditions.

We calculated the phase of the individual ASSRs as the mean angle of the complex 40-Hz Hilbert transformed waveforms in the same 250 ms to 400 ms response interval used to calculate the amplitude. The phase angle was used as an indicator of response latency. The latency can be estimated as the group delay, which is defined as the first derivative of the phase by frequency, latency=dφ/dω(Kuwada et al., 1986; Ross et al., 2000; L. Wang et al., 2021). The absolute latency of the 40-Hz response is larger than the 25-ms period, causing a phase delay which is ambiguous by multiples of 25 ms. However, we expected the latency differences between conditions and age groups to be shorter than half the 25-ms period. In this case, the latency calculation simplifies to $\Delta \text{t}=\Delta \varphi /2\text{π}f_{m}$, with *f_m_* equal to 40 Hz.

### 2.6 MRS data

We used a MEGA-PRESS (Mescher et al., 1998) protocol to measure GABA levels in 25 × 25 × 25 mm cubic volumes of interest centered on the left and right primary auditory cortex. Pre-processing and quantification were performed with Matlab (The Mathworks, Natick, USA) using the Gannet MRS toolbox (Edden et al., 2014) with some modifications. Details of the MRS acquisition, pre-processing, quantification, and tissue correction have been reported previously (Dobri & Ross, 2021; Dobri et al., 2022).

We analyzed three MRS measures focusing on different aspects of GABA because how different GABA measures relate to brain function remains unclear (Maes et al., 2018; Stagg et al., 2011). Two GABA measures were referenced to the internal H_2_O signal. The first was the total amount of GABA in the volume of interest, corrected for tissue differences in relaxation times and MR-visible H_2_O, calculated according to Equation 4 from Harris et al. (2015). The second was the concentration of GABA relative to H_2_O in the gray matter (GM), calculated according to Equation 5 from Harris et al. (2015). The third GABA measure was referenced to the internal creatine (Cr) signal and was calculated by multiplying the GABA/Cr signal ratio by 6 mMol/L, the approximate concentration of Cr in the temporal region (Christiansen et al., 1993). The GABA/Cr ratio represents the concentration of GABA in brain matter without incorporating any assumptions underlying the sophisticated tissue correction process. Therefore, comparing the GABA/Cr ratio with the GM GABA concentration is valuable for checking the reliability of the tissue correction process. Referencing GABA to both H_2_O and Cr also helps to establish that it is GABA, not the reference signals, that is driving any observed effects, as brain H_2_O and Cr content change in aging (Cleeland et al., 2019; Neeb et al., 2006; Papadaki et al., 2019).

### 2.7 Statistical analysis

We studied group effects of age and hemispheric effects on the phase and amplitude of the 40-Hz responses using mixed-measures ANOVAs with the within-participant factors “hemisphere” (left and right) and “condition” (quiet and noise) and the between-group factor “age” (young and older). ANOVAs were performed using the ezANOVA (Lawrence, 2016) package in R (R Core Team, 2020). We evaluated group mean and hemispheric effects post hoc using two-sample and paired t-tests, respectively. t-Tests were performed in Matlab using the *ttest* and *ttest2* functions. We calculated the 95% confidence interval (CI_95_) for the means using the *ttest* function. We also quantified the lateralization of ASSR amplitudes using the laterality index (LI), where $\text{LI=}\frac{\left(\text{Amplitude Right}\right)\text{-(Amplitude Left)}}{\left(\text{Amplitude Right}\right)\text{+(Amplitude Left)}}$.

In addition to analyzing differences in the group mean, we hypothesized that effects of aging would be expressed as correlations within the older group. Therefore, we used age as a continuous variable and modeled the ASSR amplitudes with a linear model using the *fitlm* function in Matlab. We also used linear modeling to investigate the association between the 40-Hz ASSR amplitudes and the auditory performance measures of hearing loss and speech-in-noise loss, as well as the relationship between the 40-Hz ASSR amplitudes and the three measures of auditory cortex GABA. Linear modeling was performed separately in each age group. Because the ASSR is generated through large-scale network interactions and tightly correlated across the cortex (e.g., Tada et al., 2021), we averaged the 40-Hz ASSRs across the left and right hemispheres for the linear modeling. However, we analyzed the GABA measures separately for each hemisphere because GABA acts locally.

### 2.8 Mediation analysis

A mediation analysis (Kenny et al., 2003; Shrout & Bolger, 2002) provided insights into the associations between the multiple univariate linear models analyzed in this study. The mediation analysis tests whether the relationship between two variables A and B may be transmitted through a third variable M, the mediator. Specifically, we tested whether the effect of age on SIN loss was mediated by auditory cortex GABA concentration and the 40-Hz ASSR amplitude and whether an effect of the gamma amplitude on SIN loss was mediated by GABA. We used the CanLab mediation package for Matlab (freely available at https://github.com/canlab/MediationToolbox), which uses bootstrap resampling for testing the significance of the mediation model.

## 3 Results

### 3.1 Auditory abilities

**Fig. 1.**
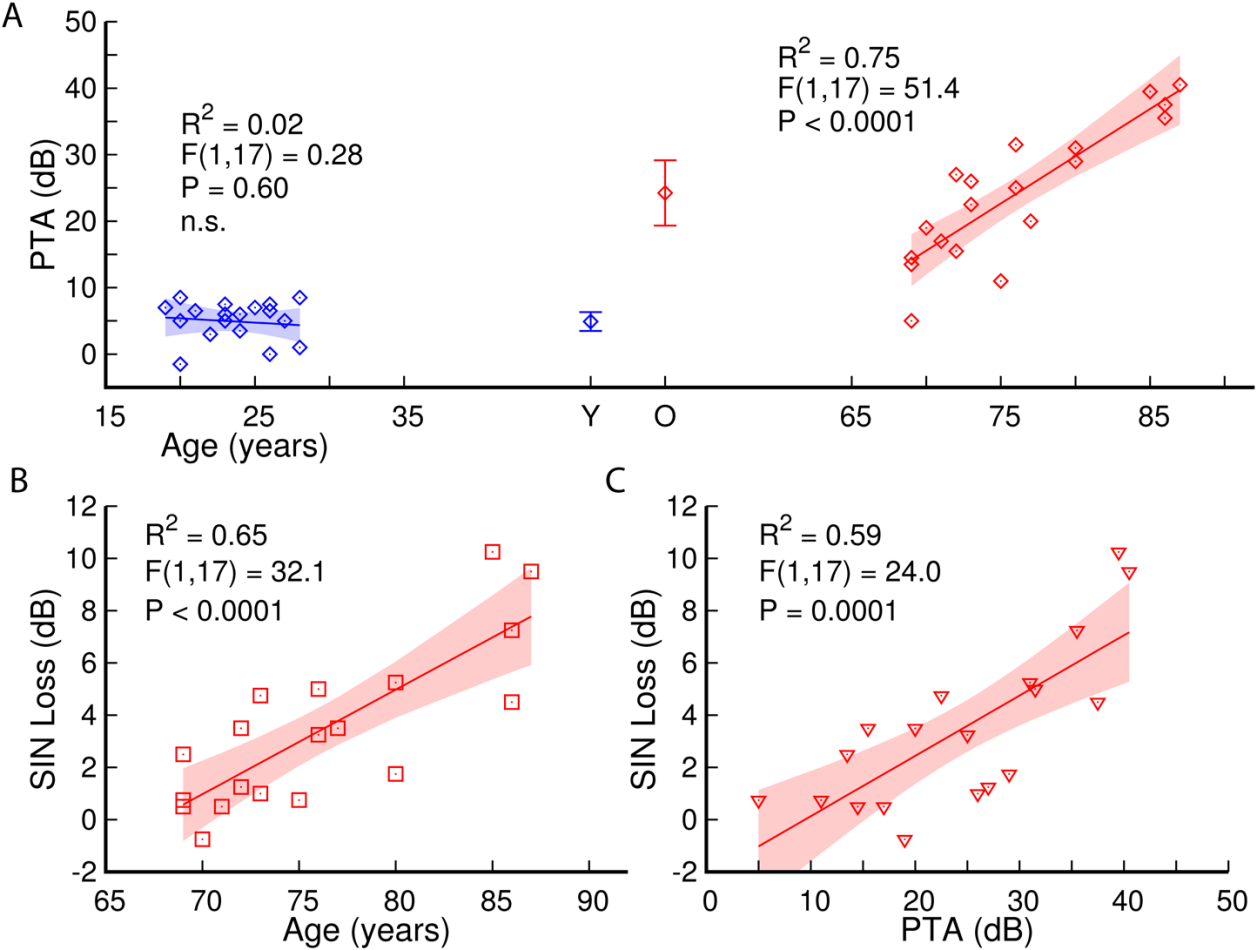
Pure-tone averaged (PTA) hearing thresholds and speech-in-noise (SIN) loss. (A) Peripheral hearing loss increased with age, as indicated by the non-overlapping 95% confidence intervals (CI) for the mean in the young (Y) and older (O) groups, and the significant correlation with age in the older group. (B) SIN loss significantly increased with older age. (C) Correlation between hearing loss and SIN loss. The shaded areas represent the 95% CIs for the linear models.

Figure 1 illustrates the effects of age on hearing loss and SIN loss. Older participants showed elevated hearing thresholds, especially at high frequencies. In the group mean, PTA was 20.6 dB larger in the older than younger participants (t(36) = 7.86, P < 0.0001). The group difference in PTA was 12.9 dB at 500 Hz, that is*,* the frequency range of the AM stimulus. Hearing thresholds increased by 15 dB per decade within the older group (R^2^ = 0.75, F(2,17) = 51.4, P < 0.0001, Fig. 1A). Also, SIN loss increased with age (R^2^ = 0.65, F(2,17) = 32.1, P < 0.0001, Fig. 1B) and was positively correlated with hearing loss (R^2^ = 0.59, F(2,17) = 24.0, P = 0.0001, Fig. 1C).

### 3.2 Source locations

Dipole models were successfully fitted for all participants. Grand-mean dipole coordinates in MNI space were -48 mm (left), -17 mm (parietal), and 8 mm (superior) in the left hemisphere, and 48 mm (right), -20 mm (parietal), and 8 mm (superior) in the right hemisphere. The source coordinates coincided with Heschl’s Gyrus on a standardized atlas, therefore localized to the primary auditory cortex (Penhune et al., 1996). An ANOVA on the dipole coordinates with the factors “age group,” “noise condition,” and “hemisphere” revealed a main effect of “hemisphere” (F(1,36) = 40.5, P < 0.0001). Sources were located 4.6 mm more anterior in the right hemisphere than the left, corroborating a well-documented hemispheric asymmetry of primary auditory cortices (Penhune et al., 1996; Rademacher et al., 2001; Warrier et al., 2009). An “age group” × “hemisphere” interaction (F(1,36) = 6.38, P = 0.016) occurred because of a significant asymmetry in the young (7.0 mm more anterior, t(18) = 8.60, P < 0.0001) but not the older group (2.0 mm more anterior, t(18) = 1.97, P = 0.065, n.s.). However, the effect of “age group” was not evident (F(1,36) < 3.4, P > 0.07, for all coordinates). The Euclidian distance between the group mean source locations was 5.0 mm (CI_95_ = [2.3 mm, 8.4 mm]). A small effect of “noise condition” was found (F(1,36) = 5.78, P = 0.021); with concurrent babble noise, sources were located 1.4 mm more anterior (t(1,37) = 2.11, P = 0.042) and 1.5 mm more medial (t(1,37) = 2.34, P = 0.025). Despite the slight source localization differences between conditions, we assume that gamma-band ASSR originates from spatially overlapping generators.

### 3.3 40-Hz ASSRs

**Fig. 2.**
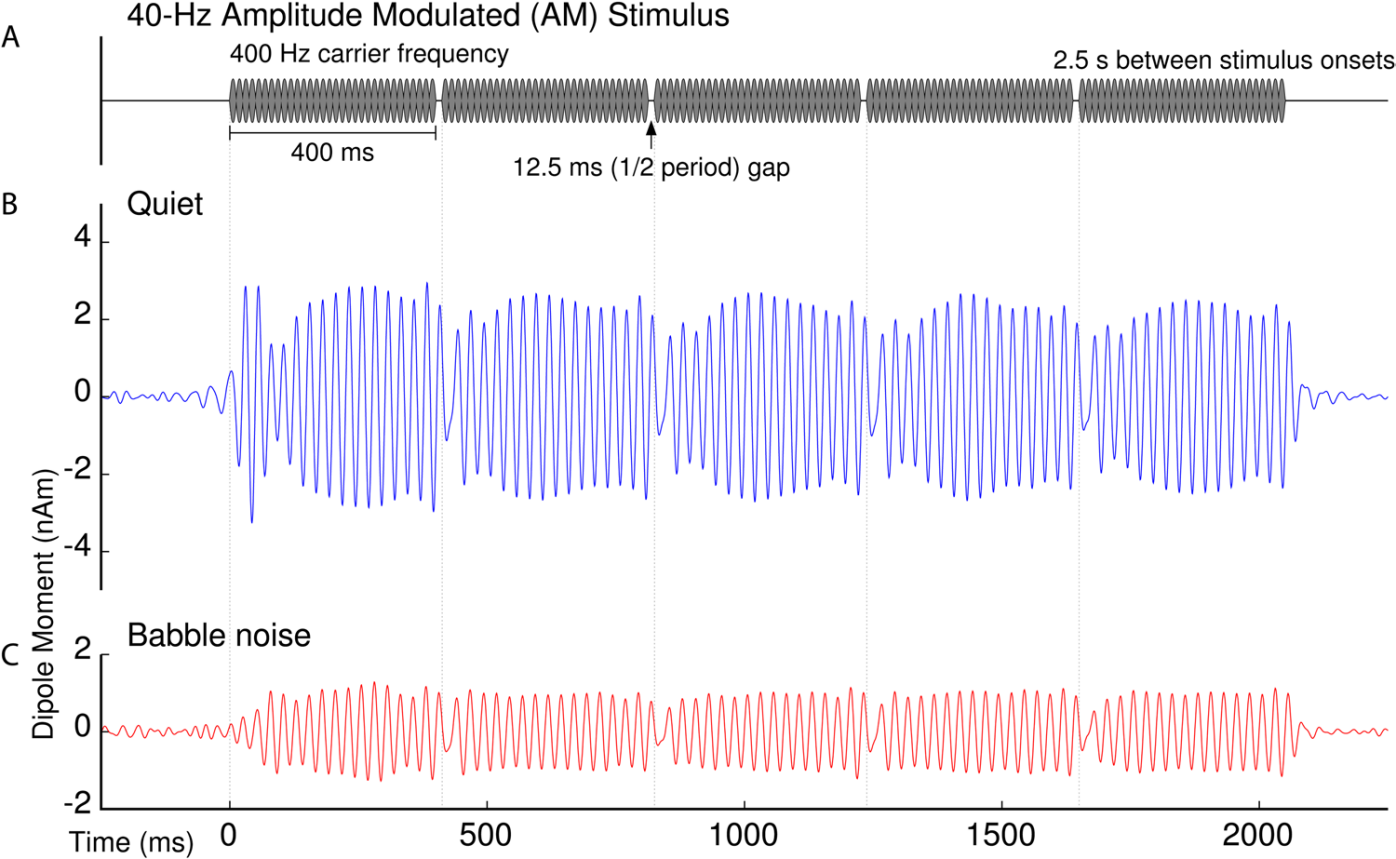
Grand-mean ASSR responses. (A) Time course of the 40-Hz AM stimulus. (B) Waveform of the grand-averaged 40-Hz dipole source activity observed without noise, averaged across left and right, and bandpass filtered between 28 Hz and 56 Hz. (C) ASSR in the multi-talker babble noise condition filtered with the same bandpass filter. The amplitude was substantially attenuated in noise compared to the quiet condition.

Significant ASSR responses were observed in all participants in quiet and with concurrent noise. In all but one case, the ASSR amplitude was larger than the largest sample of bootstrap resampling (n = 1,000) with randomized phase. The exception was for one participant in the noise condition, where 2.6% of samples under the null distribution were larger than the observed ASSR amplitude. The grand mean 40-Hz ASSRs are illustrated in Figure 2 in relation to the time course of the 40-Hz AM stimulus. The ASSR waveforms exhibited a clear reset following each gap in the AM stimulus. The ASSR waveform elicited in the quiet condition showed a distinct transient response with the stimulus onset. Such a transient response was largely absent for the noise condition because sound presentation began with the onset of the babble noise, 500 ms prior to the AM sound onset. The presence of babble noise substantially attenuated the ASSR amplitude. The babble noise also affected the temporal dynamics of the ASSR, evident from the buildup period after the AM sound onset and following each gap. These condition-dependent waveform characteristics were consistent with a previous study (Ross & Fujioka, 2016), in which the 40-Hz ASSR in quiet was separated into different components that were differently affected by the babble noise.

### 3.4 Effects of age and stimulus condition on the ASSR phase

**Fig. 3.**
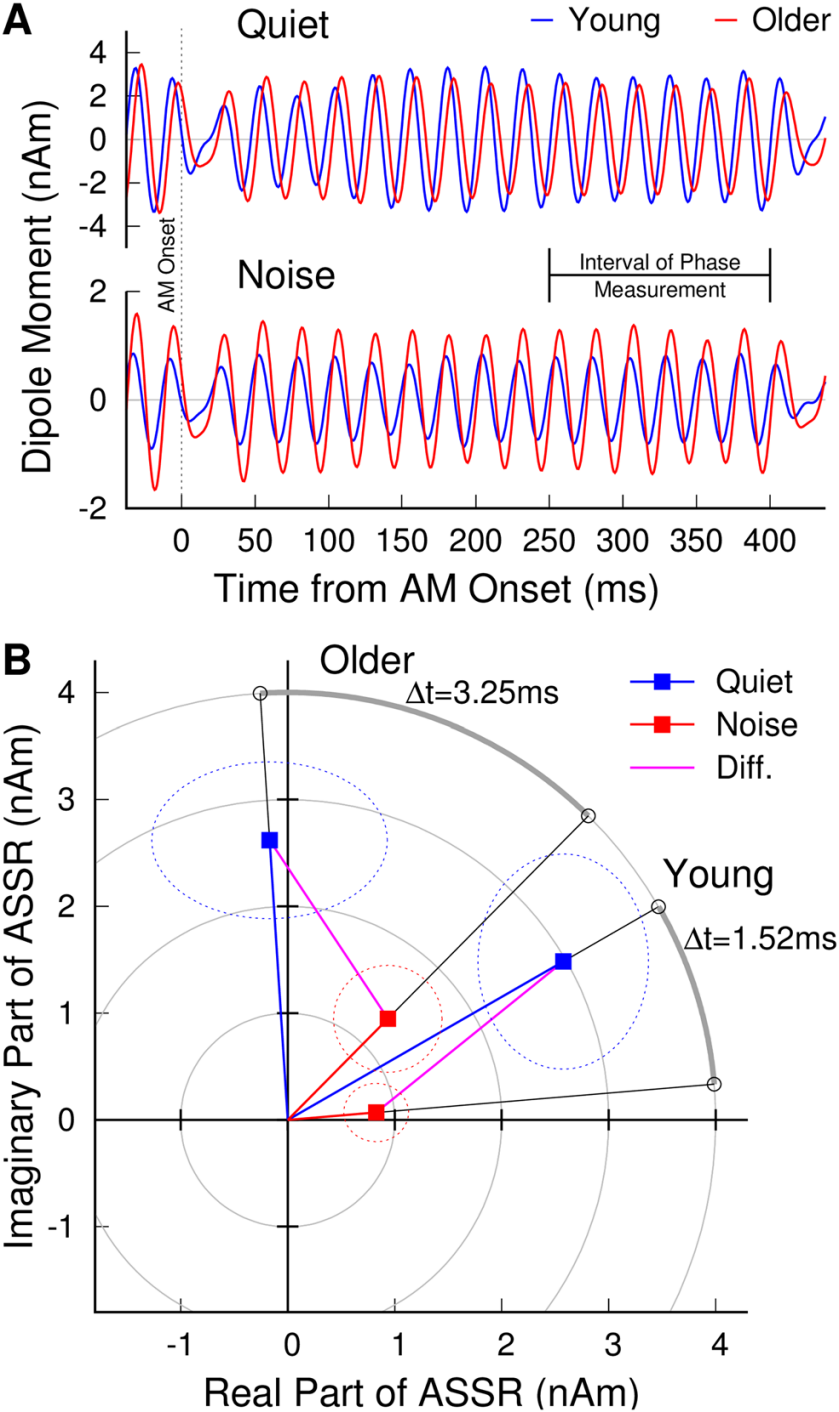
ASSR latencies in young and older adults. (A) The group-mean time series of 40-Hz responses are delayed in the older compared to the young group. (B) Group-mean ASSRs averaged across hemispheres represented in the complex plane. Rotation of the phase angle demonstrates longer latency in the older group compared to young and later response in quiet than in noise. Dashed ellipses denote the 95% CIs in the mean real and imaginary components.

Visual inspection of the group-mean waveforms (Fig. 3A) revealed that the ASSR was delayed in the older compared to the young group. We analyzed differences in the response latency Δt between stimulus conditions and age groups based on phase differences Δϕ of the complex ASSR, using the relationship $\Delta \text{t}=\Delta \varphi /2\text{π}f_{m}$ with the stimulus frequency *f_m_* = 40 Hz. The ASSR phase angles are illustrated with the group mean ASSRs in the complex plane in Figure 3B. The ANOVA on the 40-Hz ASSR phase in the quiet and noise conditions revealed effects of the between-group factor “age” (F(1,36) = 24.3, P < 0.0001, η^2^ = 0.36) and the within-participant factor “condition” (F(1,36) = 39.9, P < 0.0001, η^2^ = 0.14). Group-mean latencies were prolonged by 4.39 ms in older compared to young adults (t(36) = 4.9, P < 0.0001). The grand mean latency was 2.38 ms shorter in the noise compared to the quiet condition (t(37) = 5.98, P < 0.0001). An “age” × “condition” interaction (F(1,36) = 5.37, P = 0.026) occurred because the latency difference between stimulus conditions was more pronounced (t(36) = 2.29, P = 0.028) in older (Δt = 3.25 ms, t(18) = 6.04, P < 0.0001) than young adults (Δt = 1.52 ms, t(18) = 3.09, P = 0.0064). The shorter latency in the noise condition may seem counterintuitive because it has been previously reported that response latencies increase with reduced stimulus intensity (Roberts et al., 2000). Therefore, if the latency were related to the audibility of the stimulus, one would expect it to increase in noise. However, the shorter latency in the noise condition could reflect early sensory activity, whereas the response in quiet may be dominated by higher-order processing. The larger latency difference in older adults is consistent with greater aging-related delays for higher-order than early responses (Ross et al., 2007).

### 3.5 Effects of age and stimulus condition on 40-Hz ASSR amplitudes

**Fig. 4.**
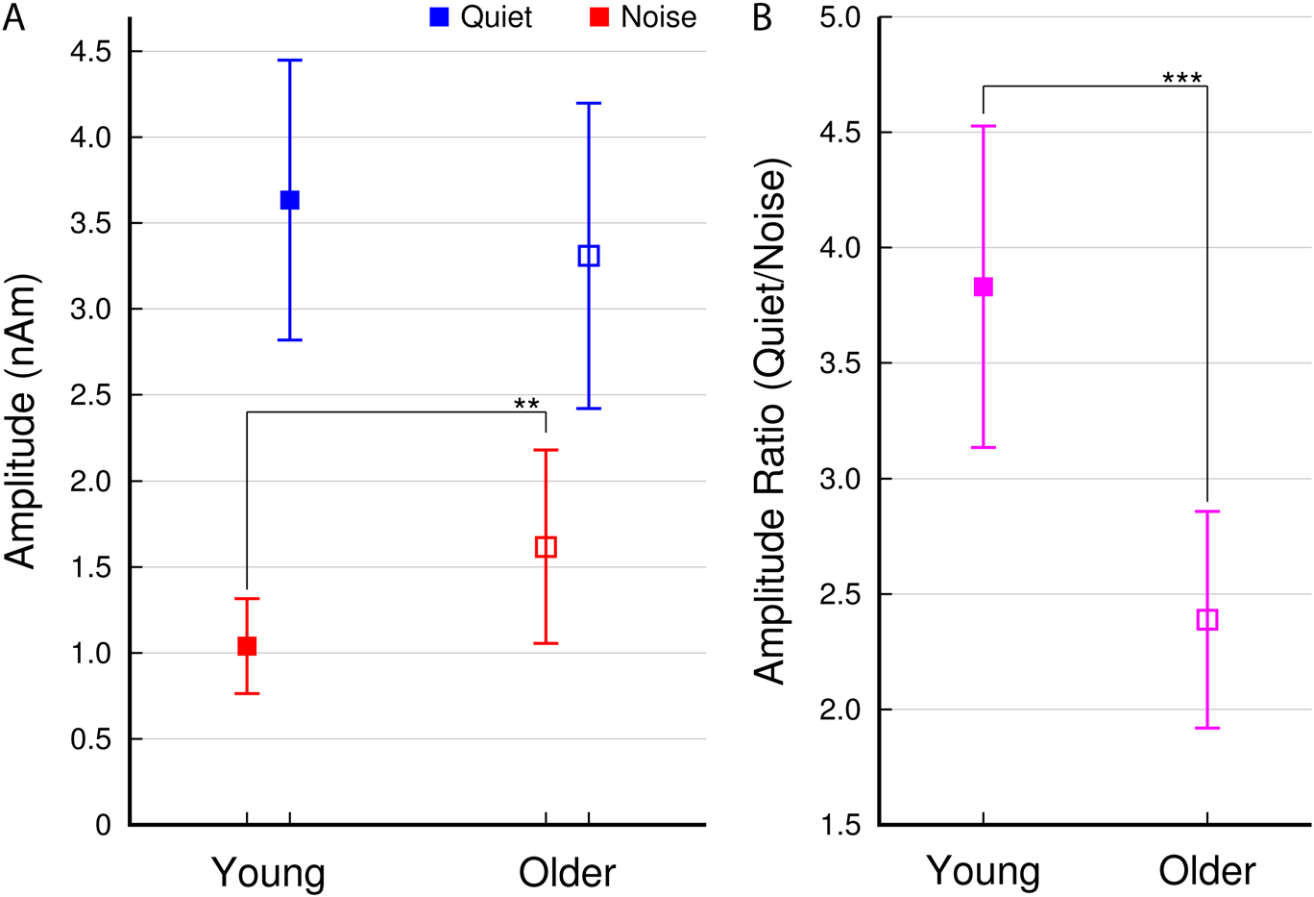
(A) Group-mean 40-Hz ASSR amplitudes in the quiet and noise conditions. (B) Group-mean amplitude ratios between conditions (quiet /noise). Error bars denote the 95% CIs. Statistical significance is indicated by **: P < 0.01, ***: P < 0.001.

Group-mean 40-Hz ASSR amplitudes are plotted in Figure 4A. The ANOVA on the amplitudes of the 40-Hz ASSRs revealed a main effect of “condition” (F(1,36) = 118.8, P < 0.0001, η^2^ = 0.37). As expected, the amplitudes were more prominent in quiet (3.51 nAm) compared to noise (1.33 nAm, t(37) = 10.1, P < 0.0001). There was no main effect of “age” (F(1,36) = 0.12, P = 0.73, n.s.); thus, the group mean ASSR amplitude in older adults (2.22 nAm) was not significantly different from the young group (2.26 nAm, t(36) = 0.1, P = 0.9). However, an “age” × “condition” interaction was significant (F(1,36) = 5.27, P = 0.028). In quiet, the amplitudes were not different between both groups (young: 3.66 nAm; older: 3.37 nAm; t(36) = 0.53, P = 0.6), while in the noise condition, the amplitudes were larger in the older group (young: 1.04 nAm, older: 1.63 nAm, t(36) = 2.90, P = 0.006). Concurrent noise reduced the ASSR by 71.2% in the young but only 50.4% in the older group (t(36) = 3.65, P < 0.0001). That noise attenuated the ASSR amplitudes in older adults to a lesser extent than in young seems counter-intuitive because one could assume that older people would experience more interference from noise. An alternative explanation is that older people did not benefit as much from the absence of noise as younger people did.

**Fig. 5.**
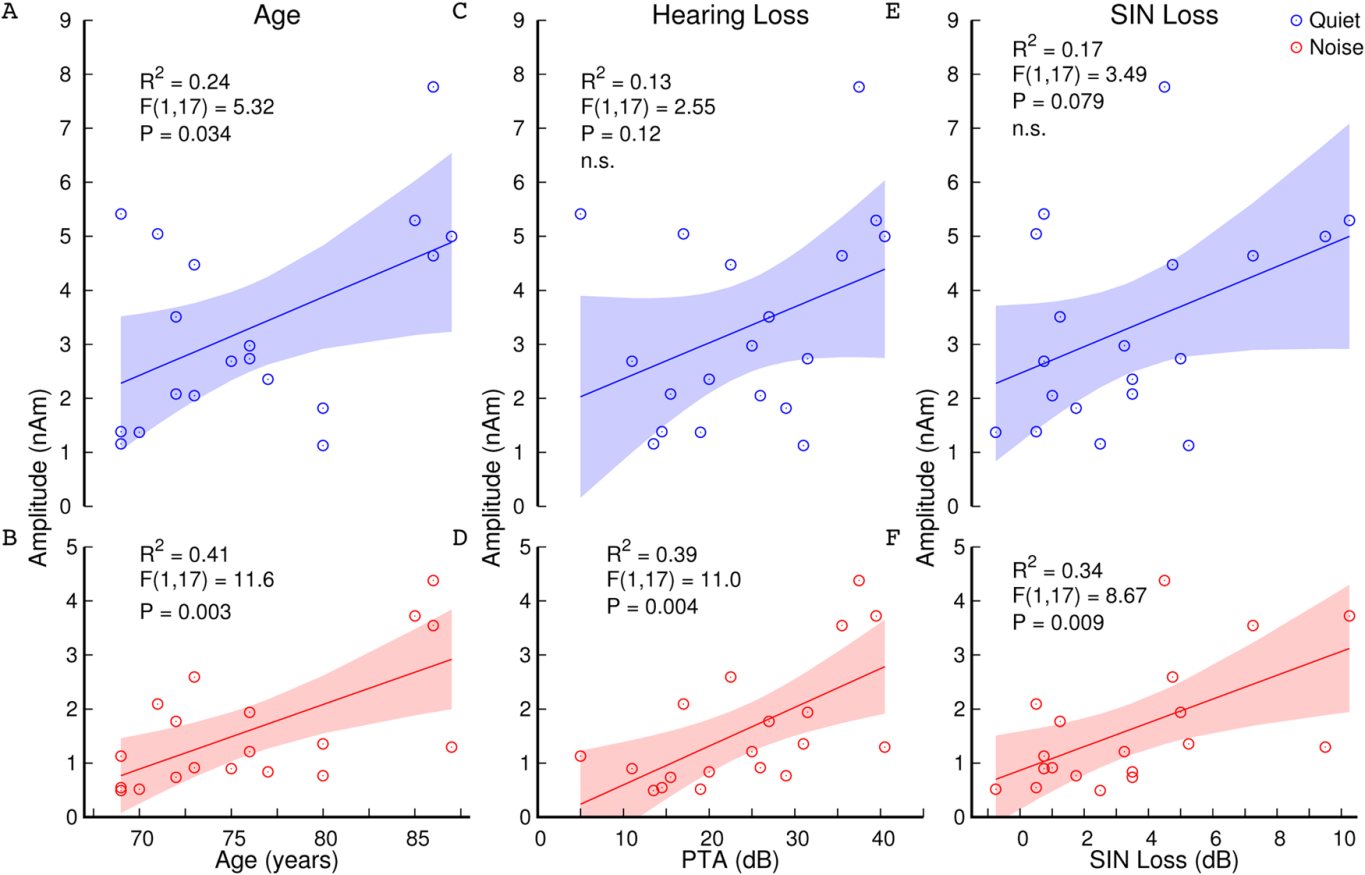
Linear models of the 40-Hz ASSR amplitudes in the older group as functions of age, hearing loss, and SIN loss. Amplitudes were significantly correlated with age in the quiet (A) and multi-talker babble noise (B) conditions. Amplitudes in the noise condition were also correlated with hearing loss (D) and SIN loss (F); however, linear models with auditory performance were not significantly different from constant in the quiet condition (C & E). Shaded areas represent the 95% CIs for the linear models.

Group mean differences may not wholly describe the effects of aging on the ASSR amplitudes because the onset of aging effects may occur later in life and become apparent as amplitude changes within the older group. Therefore, we modeled the ASSR amplitudes as a linear function of age for the older group. With and without noise, ASSR amplitudes increased with age within the older group, as demonstrated by the positive correlations in Figures 5A and 5B. The slope of aging-related ASSR increase was similar for the quiet (0.16 nAm/year, CI_95_ = [0.03, 0.29] nAm/year) and the noise condition (0.12 nAm/year, CI_95_ = [0.05, -0.19] nAm/year). Because of the approximately parallel regression lines (Fig. 5), the difference in ASSR amplitudes between the conditions did not significantly change with age (R^2^ = 0.02, F(2,17) = 0.31, P = 0.58, n.s.). However, the relative amplitude increase was steeper in the noise condition in which amplitudes were generally smaller. The fraction to which concurrent noise attenuated the ASSR amplitude was 33% at age 65 and increased to 63% at age 85. However, the linear model for this trajectory did not reach significance (R^2^ = 0.15, F(2,17) = 3.36, P = 0.084, n.s.).

### 3.6 Hemispheric effects on 40-Hz ASSRs

We expected that ASSR amplitudes would be more pronounced in the right than the left hemisphere, as ASSR lateralization has been previously reported (Ross et al., 2005). Furthermore, we were interested in whether age and noise condition affected the ASSR lateralization. The ANOVA on the 40-Hz ASSR amplitude revealed a main effect of “hemisphere” (F(1,36) = 18.0, P = 0.0005) and a “hemisphere” × “condition” interaction (F(1,36) = 25.4, P < 0.0001) but no “hemisphere” × “age” interaction (F(1,36) = 0.38, P = 0.54, n.s.). For both age groups, the ASSR amplitudes were right-lateralized in the quiet condition (LI = 0.13, CI_95_ = [0.07, 0.19]), but not with concurrent noise (LI = 0.03, CI_95_ = [-0.04, 0.10]).

### 3.7 40-Hz amplitude and auditory abilities

Figure 5 shows scatter plots and linear models of the ASSR amplitude dependence on age, hearing loss, and SIN loss within the older group. Larger 40-Hz ASSR amplitudes in the noise condition were associated with increased severity of hearing loss (R^2^ = 0.39, F(1,17) = 11.0, P = 0.004; Fig. 5D) and SIN loss (R^2^ = 0.34, F(1,17) = 8.67, P = 0.009; Fig. 5F).

### 3.8 Relationship between GABA and auditory abilities in aging

**Fig. 6.**
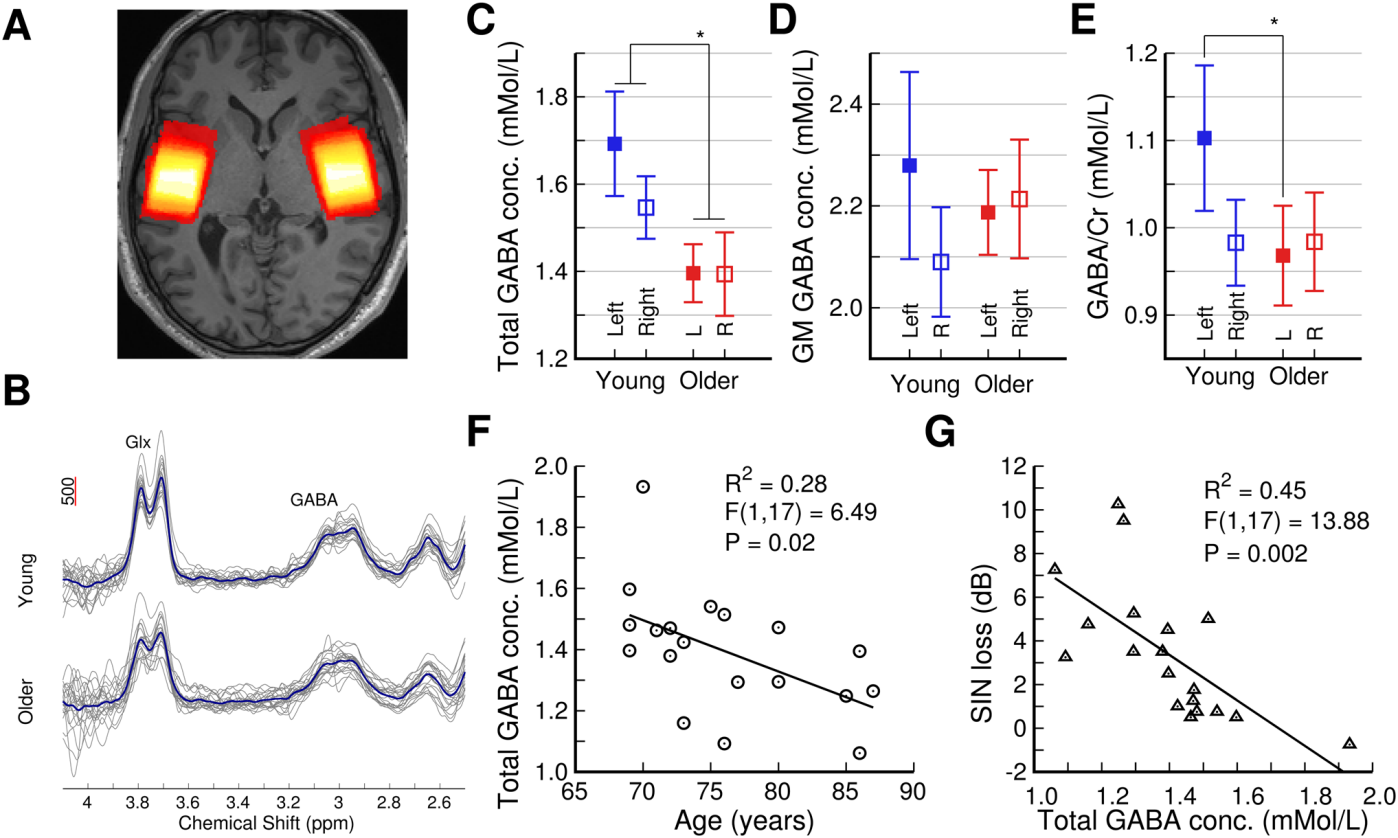
Summary of GABA changes in aging and their relationship to auditory performance, previously reported in Dobri and Ross (2021). (A) Overlay of MRS volumes of interest for all participants on an individual structural image. White represents more overlap, red less. (B) Individual (gray) and group-mean (blue) right hemisphere GABA spectra. (C, D, & E) Group-mean total GABA, GM GABA, and GABA/Cr, respectively. Error bars represent the 95% CIs in the mean. Statistical significance is indicated by *: P < 0.05. (F) Correlations between age and the right hemisphere total GABA level in the older group. (G) Correlation between SIN loss and the right hemisphere total GABA level in the older group.

We previously reported the relationships between auditory cortical GABA, hearing loss, and SIN loss for the same participants as in the current study (Dobri & Ross, 2021). In summary, the group-mean total GABA levels were lower in the older than the young group for both the left and right hemispheres (Fig. 6C). The left hemisphere GABA/Cr ratio was also lower in the older group (Fig. 6E), whereas there was no evidence of an age group difference in the GM GABA concentration (Fig. 6D). Within the older participants, only the total GABA level in the right hemisphere was significantly associated with age (negatively, see Fig. 6F) and SIN loss (Fig. 6G), but not with hearing loss.

### 3.9 GABA and 40-Hz amplitudes

**Fig. 7.**
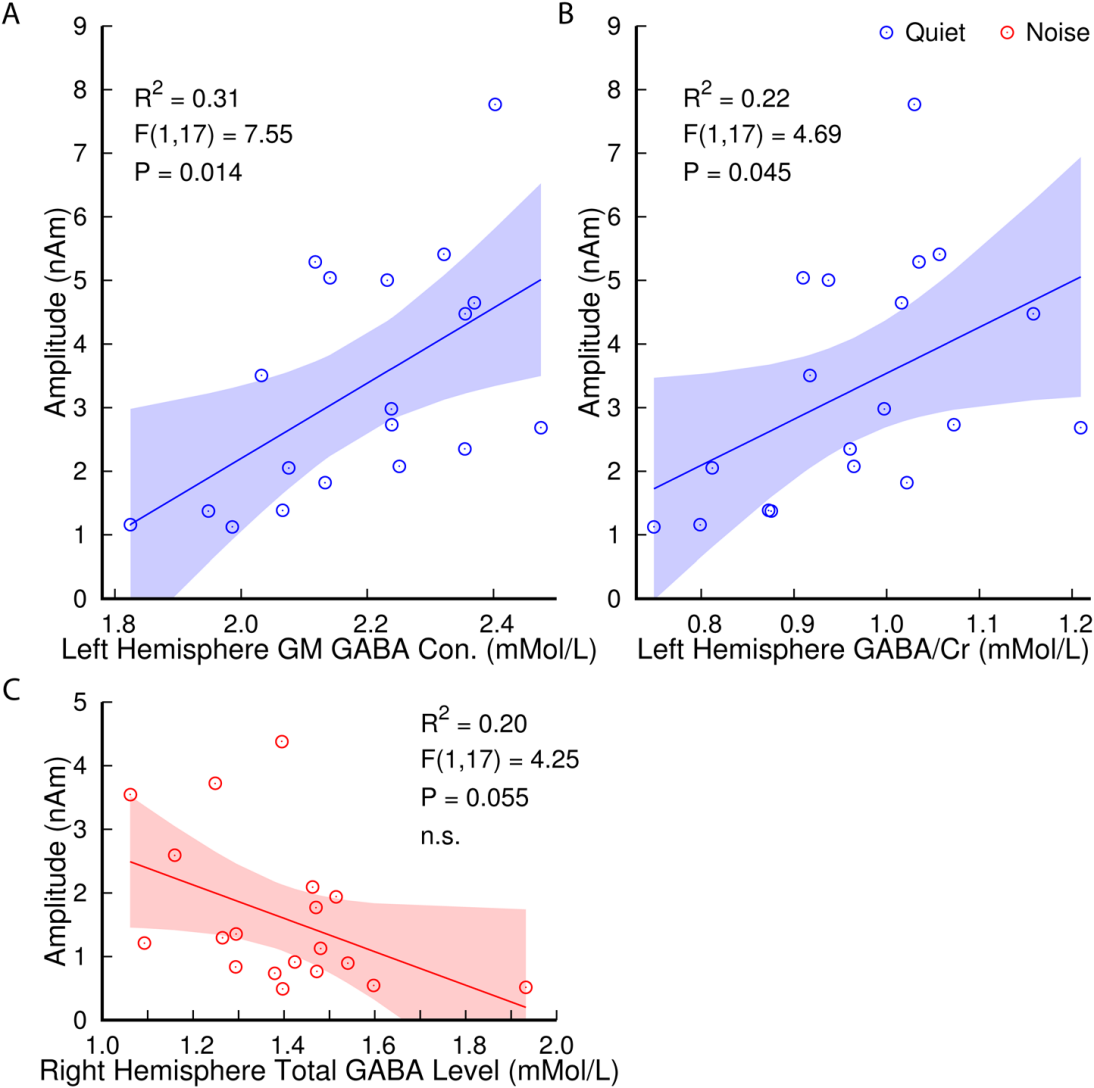
Correlations between the 40-Hz ASSR amplitudes averaged across hemispheres and GABA in the older group. The amplitude in quiet was positively correlated with the GM GABA concentration (A) and the GABA/Cr ratio (B) in the left auditory cortex. There was a trend toward a negative correlation between the amplitude in noise and the total GABA level in the right auditory cortex (C). Shaded areas represent the 95% CIs for the linear models.

Scatter plots and linear models in Figure 7 illustrate the relationship between auditory cortex GABA levels and ASSR amplitudes in the older group. The left hemisphere GM GABA concentration was positively correlated with the 40-Hz ASSR amplitudes in quiet (R^2^ = 0.31, F(1,17) = 7.55, P = 0.014; Fig. 7A). There was also a positive correlation between the left hemisphere GABA/Cr ratio in the older group and the 40-Hz ASSR amplitudes in quiet (R^2^ = 0.22, F(1,17) = 4.69, P = 0.045; Fig. 7B). However, with concurrent noise, there was only a tendency for a correlation between GM GABA and ASSR amplitudes (R^2^ = 0.15, F(1,17) = 3.1, P = 0.097). There was a tendency for a negative correlation between the total GABA level in the right hemisphere and the ASSR amplitude in the noise condition (R^2^ = 0.20, F(1,17) = 4.25, P = 0.055, n.s.; Fig. 7C), consistent with the directions of the corresponding relationships with age and SIN loss. Within the young group, linear regressions between GABA and gamma amplitudes were not significant (P > 0.05).

### 3.10 Mediation analysis

**Fig. 8.**
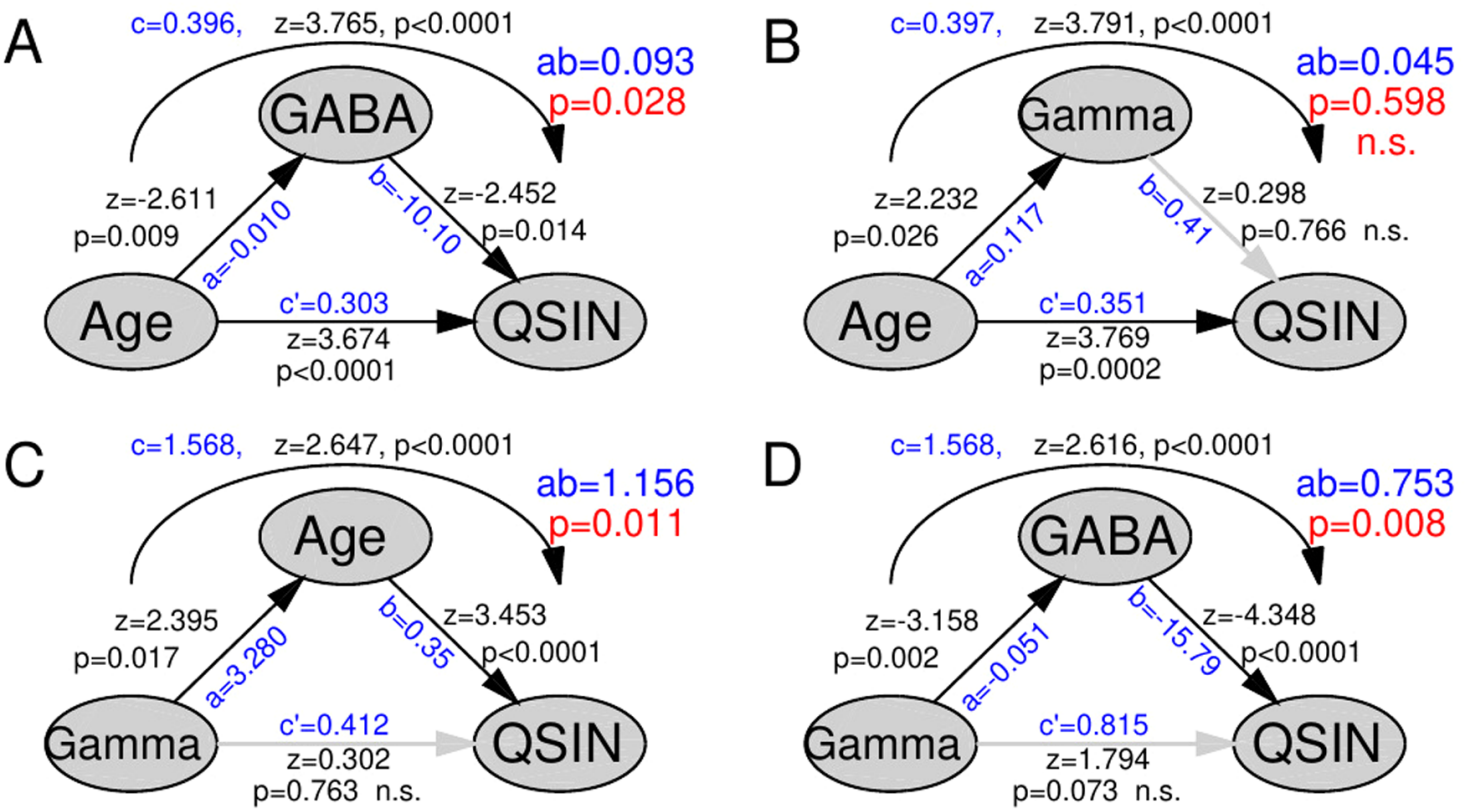
Mediation analysis of the associations between age, speech-in-noise loss (QSIN), right auditory cortex total GABA level (GABA), and noise-condition 40-Hz ASSR (gamma) amplitudes. (A) The effect of age on SIN loss was mediated by GABA. The labels along the paths indicate the effect sizes (blue) and bootstrapped z-scores and p-values (black). The p-value for the mediation effect is included at the top right in red. (B) The effect of age on SIN loss was not significantly mediated by the ASSR amplitude. (C) Instead, age predominantly mediated the effect of gamma on SIN loss. (D) The effect of the 40-Hz ASSR amplitude on SIN loss was predominantly mediated by GABA.

First, we corroborated the previously reported finding that auditory cortex GABA level mediated the effect of age on SIN loss (Dobri & Ross, 2021) for the n = 19 participants in this study (Fig. 8A). The indirect path of decreasing GABA with increasing age and the positive correlation between GABA and SIN loss better explained the increasing SIN loss with increasing age than the direct effect. Second, a corresponding analysis was applied to the ASSR amplitude observed in the noise condition. Although the ASSR amplitude positively correlated with age and SIN loss, the correlation between age and SIN loss was not mediated by the gamma ASSR amplitude (Fig. 8B). Instead, age predominantly mediated the effect of the gamma amplitude on SIN loss (Fig. 8C). Third, we investigated whether GABA was involved in the association between gamma and SIN loss. The mediation analysis showed that the path of linear correlations between gamma and GABA and GABA and SIN loss better explained the association between gamma and SIN loss than the direct path (Fig. 8D).

## 4 Discussion

**Fig. 9.**
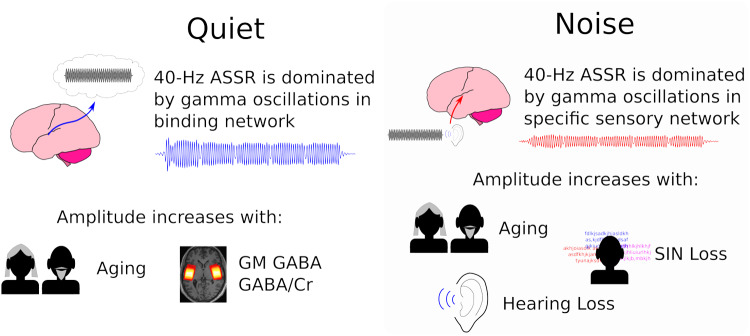
Summary and interpretation of the findings. The responses significantly differed between listening conditions, indicating the 40-Hz ASSR contains two functionally distinct components. We attribute these components to activity in specific sensory and higher-order binding thalamocortical networks. In quiet, the longer delay and increased amplitude indicate the ASSR is dominated by oscillations in the binding network. This gamma activity underlies the representation of the AM stimulus as a perceptual object. The babble noise disrupts 40-Hz synchrony in the binding network, so the response in noise is dominated by oscillations in the specific sensory network. This gamma activity represents the primary acoustic features of the AM stimulus. The 40-Hz amplitudes in both networks increased in aging. Larger amplitudes in quiet were associated with higher GABA concentrations in brain matter. Larger amplitudes in noise were associated with increased hearing loss and poorer SIN performance.

We recorded 40-Hz ASSRs with MEG with and without concurrent multi-talker babble noise in young and older healthy adults. Auditory cortex GABA concentrations were estimated in the same participants using MRS. Within the older group, we correlated the gamma-band ASSR and GABA measures with SIN loss and age. ASSR latencies were shorter in the noise than quiet condition, which will be discussed as indicating multiple components of gamma oscillations: an earlier sensory component which dominated the response in the noise condition and a later, higher-order component which was more prominent in quiet. Figure 9 illustrates how the two ASSR components changed in aging and their relationship to SIN and hearing loss and GABA levels. ASSR amplitudes increased with age, SIN loss, and hearing loss. Moreover, the ASSR amplitude was positively correlated with the auditory GABA concentration. We will interpret larger 40-Hz ASSR amplitudes as increased gamma synchrony because the MEG signal reflects the synchronous activity of large neuronal ensembles.

### 4.1 Dissociation between sensory and higher-order components of the 40-Hz ASSR

Concurrent multi-talker babble noise substantially attenuated the 40-Hz ASSR amplitude in both age groups. The experimental design was informed by previous work (Ross & Fujioka, 2016) that separated 40-Hz oscillations in spatially overlapping thalamocortical networks involved in representing elementary sensory features and higher-order object representation, that is, feature binding (Jones, 2009). In quiet, 40-Hz oscillations in both the sensory and binding networks were synchronized with the 40-Hz AM stimulus. In noise, the 40-Hz ASSR amplitude was substantially reduced because the concurrent multi-talker babble noise continually disrupted synchrony between the AM stimulus and 40-Hz oscillations in the binding network. In contrast, the noise had little effect on oscillations in the sensory network, leaving them to dominate the ASSR in the noise condition.

Our analysis of the 40-Hz ASSR phase in the different stimulus conditions further informs the interpretation of the ASSR components. Concurrent noise reduced the ASSR latency compared to the response in quiet. Previous studies showed that background noise attenuated the amplitude and delayed the peak latency of auditory responses (Billings et al., 2009; Kim et al., 2012). However, in our study, the babble noise was filtered to avoid spectral overlap with the AM sound, ensuring that interference occurred in the central rather than peripheral auditory system. Therefore, the effect was comparable to that of contralateral masking, which shortened latencies (Salo et al., 2003). The noise likely decreased the latency of the 40-Hz ASSR because it affected higher-order processing with prolonged latency, leaving the early sensory component relatively unaffected.

Aging also affected the 40-Hz ASSR phase. In both stimulus conditions, the phase lag of the ASSR relative to the stimulus increased with advancing age, corroborating the findings of an aging-related latency increase from our previous study (Ross & Fujioka, 2016). Consistently, aging-related increases have been reported for the P2 latency (Alain & McDonald, 2007; Tremblay et al., 2003) and the middle latency (Woods & Clayworth, 1986) auditory-evoked responses. A recent study found that the 200-ms-latency peak of the temporal response function to speech was also delayed in older adults (Karunathilake et al., 2023). Most strikingly, we found an interaction between age and stimulus condition on the 40-Hz ASSR phase: the aging-related latency was more prolonged in the quiet than in the noise condition. An aging-related latency increase can be partially explained by changes in the peripheral auditory system, such as increased travel time of the soundwave in the cochlea (Ramotowski & Kimberley, 1998). Moreover, changes in gray and white matter throughout the auditory pathway cause slower neural conduction in aging, resulting in a cumulative delay of auditory brain responses (Price et al., 2017); thus, later responses are more delayed than early responses (Ross, 2013). Therefore, aging-related changes in the auditory system caused more of a delay in the higher-order response dominating the 40-Hz ASSR quiet than in the sensory response, which is more prominent in the ASSR in the noise condition. Though we did not analyze the functional significance of the delay in the current study, a delayed gamma response has been found in Alzheimer’s patients compared to healthy aging (Başar et al., 2016), suggesting it may be relevant for aging-related impairments in cortical processing.

### 4.2 Aging-related changes in 40-Hz ASSR amplitudes

In the group mean, the 40-Hz amplitudes were not different between young and older adults. However, within older adults, the amplitudes increased with age. This difference between comparing age-group means and analyzing the correlation within the older group highlights the importance of studying aging as a continuous process within an older population. By doing so, we were able to demonstrate the effects of aging that occur later in life and are not evident in group differences between young and older adults. Previous studies where the 40-Hz ASSR amplitude has been measured in quiet have shown either no difference in amplitude between young and older adults (Goossens et al., 2016; Purcell et al., 2004) or a larger amplitude in older adults (Farahani et al., 2020). These reports are consistent with our finding that the effects of age were more expressed within an aging population than between groups.

Human auditory responses may generally increase in aging. Broadband-evoked responses increase in aging, including the P1 (Alain et al., 2014; Pekkonen et al., 1995; Sörös et al., 2009), P50 (Brinkmann et al., 2021), and the middle latency (Amenedo & Díaz, 1998; Bidelman et al., 2014; Chambers, 1992) responses. Also, the peaks of the temporal response function to speech were larger in older compared to young adults (Karunathilake et al., 2023). Specifically in the gamma band, compared to young adults, older adults showed an increased transient auditory gamma response to tone stimuli (Christov & Dushanova, 2016; Ross, Dobri, et al., 2020; Ross, Tremblay, et al., 2020) and increased gamma power when listening to speech stimuli (Rufener et al., 2016). Thus, the increase in sensory network 40-Hz gamma synchrony may indicate that the aging central auditory system becomes more sensitive to incoming auditory stimuli.

In a previous study with monaural stimulation, we found the amplitude of the 40-Hz ASSR in quiet was larger in young compared to older adults, and there was no age group difference in noise (Ross & Fujioka, 2016), the latter possibly due to the moderate group sizes. However, in both the current and our previous study, the attenuating effect of noise on the 40-Hz ASSR was smaller in older compared to young adults. This may indicate that noise has a less disruptive impact on 40-Hz synchrony in older adults, but our findings suggest another explanation. Let’s consider the components of the 40-Hz ASSR recorded in quiet and in noise. The more minor amplitude difference between stimulus conditions indicates a reduced amplitude of binding oscillations in older adults. Therefore, our findings support our previous interpretation of an aging-related reduction in neural resources for auditory feature binding (Ross & Fujioka, 2016).

### 4.3 Gamma increases with hearing and SIN loss

Within the older group, larger amplitudes of the 40-Hz ASSR in the noise condition were associated with increasing SIN loss. Since 40-Hz gamma synchrony underlies auditory feature binding, it is reasonable to assume that stronger 40-Hz synchrony would correspond to better SIN performance. However, a study in young adults found that increased gamma was correlated with poorer performance on a tone discrimination task, indexed by reaction time (Ou & Law, 2018), indicating a larger gamma response does not necessarily correspond to better brain function. In fact, enhanced gamma activity in aging may be related to functional decline. The 40-Hz ASSR increased in Alzheimer’s patients compared to healthy, age-matched controls (Osipova et al., 2006), and this increase was related to poorer cognitive performance (van Deursen et al., 2011). To a certain extent, Alzheimer’s disease can serve as a model for accelerated aging (Leparulo et al., 2022; Toepper, 2017). Gamma increases in Alzheimer’s have also been reported for various auditory and visual stimuli (Başar et al., 2017; van Deursen et al., 2008).

We found that the relationship between increased sensory 40-Hz ASSR amplitudes and SIN loss was stronger in noise than in quiet, indicating a negative association between increased synchrony of sensory gamma oscillations and speech perception in noise. Older adults showed greater entrainment to the rhythm of attended speech (Decruy et al., 2019; Karunathilake et al., 2023; Presacco et al., 2016). However, cortical responses were also larger for the unattended competing speech (i.e., noise) (Zan et al., 2020). Thus, in aging, there would be more competition between 40-Hz oscillations involved in coding the sensory features of the background noise and the attended speech. This would make it more likely for 40-Hz oscillations for binding the speech and noise features to become synchronized, therefore bound into a single, combined auditory object. In such a case, the listener could not extract meaningful speech information, resulting in SIN loss.

Although the ASSR amplitude, SIN loss, and age were correlated, the effect of age on SIN loss was not mediated by the ASSR. Instead, age predominantly mediated the effect of the gamma amplitude on SIN loss. Thus, our analysis did not disentangle the contribution of ASSR amplitude from the effect of age on SIN loss. However, our findings would not lead us to conclude that the relationship between gamma and SIN loss is entirely explained by age. Many interacting aging-related changes contribute to SIN loss (Humes et al., 2012). It is highly likely that our sample size of 19 older adults does not provide the statistical power to accurately quantify the unique contribution of gamma synchrony. Further studies with larger sample sizes are necessary to do so.

We further found that the 40-Hz ASSR amplitude in noise was correlated with hearing loss. This could be interpreted as supporting the hypothesis that the increased sensory response is a compensatory mechanism for reduced sensory input (Stothart et al., 2013). However, a recent study of visual, auditory, and somatosensory-evoked responses found that all three increased in aging and were correlated across sensory domains, supporting the hypothesis that increased sensory responses in aging share a common cause (Alain et al., 2022). The common cause could be aging-related changes in sensory regulation mediated by the prefrontal cortex (Knight et al., 1999). Increased activity in frontal cortex areas could also be related to a compensatory mechanism (Cabeza et al., 2002; Peelle et al., 2010). Also, increased amplitudes of cortical auditory responses were more closely related to age than to hearing loss (Presacco et al., 2019), including the transient gamma-band response (Ross, Dobri, et al., 2020). Therefore, it is possible the correlation we found between the 40-Hz ASSR amplitude and hearing loss does not reflect a causal relationship but rather results from the tight correlation between hearing loss and age.

### 4.4 GABA and 40-Hz gamma amplitude

We found a positive correlation between the left-hemisphere GM GABA concentration and 40-Hz gamma amplitude in the older group for the quiet condition. Our finding that the left hemisphere GABA/Cr ratio was also positively correlated with the 40-Hz amplitude in the quiet condition supports GABA rather than H_2_O as the driver of this effect. Thus, a greater concentration of GABA in GM corresponded to increased synchronization of 40-Hz oscillations in the higher-order binding network. This positive association is consistent with how pharmacological manipulation of the GABAergic system affects the 40-Hz ASSR. In rats, increasing GABA-mediated inhibition by administering the GABA_A_ receptor agonist muscimol increased both the power and phase-locking factor of the 40-Hz ASSR (Vohs et al., 2010, 2012), and decreasing inhibition by administering the GBAA_A_ receptor antagonist bicuculine dose-dependently reduced 40-Hz ASSR power (Yamazaki et al., 2020). Similarly, in humans, tilting the excitatory-inhibitory balance in favor of inhibition through administration of the N-methyl-D-aspartate (NMDA) antagonist memantine increased the power and phase-locking of the 40-Hz ASSR in patients with schizophrenia and healthy controls (Light et al., 2017).

A positive relationship between GABA and gamma oscillations has also been found for other types of gamma activity. GABA levels in the superior temporal sulcus were correlated with gamma power in the sound-induced flash illusion, an audiovisual binding phenomenon (Balz et al., 2016). In patients with schizophrenia and healthy controls, GABA levels in the dorsolateral prefrontal cortex were positively correlated with gamma amplitude at rest and during a working memory task (Chen et al., 2014). Administering GABA_A_ receptor agonists increased resting-state gamma power across the brain (Hall et al., 2010) and increased visual cortical gamma power during a demanding visuospatial working memory task (Lozano-Soldevilla et al., 2014).

This positive relationship between GABA concentration and 40-Hz gamma synchrony can be interpreted in terms of the mechanisms of gamma generation. Synchronized gamma oscillations are established through GABA-mediated inhibition (Buzsáki & Wang, 2012; Buzsáki et al., 1983; Cardin et al., 2009; Sohal et al., 2009; Whittington et al., 1995). GABA inhibits firing in the postsynaptic cell by hyperpolarizing its membrane, bringing its potential farther from the action potential threshold. Fast-spiking GABAergic interneurons periodically inhibit excitatory pyramidal cells, defining a 40-Hz rhythm in their firing probability (Steriade et al., 1998). Increased inhibitory power, reflected in a higher GM GABA concentration, would result in greater hyperpolarization, thus more strongly restricting firing to a specific phase of the 40-Hz rhythm and increasing 40-Hz gamma synchrony.

In contrast to the quiet condition, we found no evidence of a relationship between GM GABA and the gamma amplitude in the noise condition. This supports our interpretation that the 40-Hz ASSR contains both sensory and higher-order binding components. 40-Hz oscillations in the sensory network are synchronized because of coherent input from the AM stimulus, evident in how closely the dynamics of the 40-Hz ASSR in noise follow the stimulus (Ross & Fujioka, 2016). Thus, synchrony in the sensory network is established mainly through external mechanisms. In contrast, in order to flexibly bind sensory features, synchronization of 40-Hz oscillations in the binding network must be controlled through internal mechanisms. This is apparent in the approximately 200 ms buildup time required for the 40-Hz ASSR in quiet to reach its steady state (Ross et al., 2002). Therefore, synchrony in the binding network should depend more strongly on GABA levels, consistent with our findings. This distinction between GABA’s relationship with stimulus-driven and internallygenerated synchrony may explain why GABA agonists decreased rather than increased the amplitude of the transient auditory 40-Hz response (Jääskeläinen et al., 1999) and decreased the visually evoked response but increased the gamma component (Saxena et al., 2021).

Aging affected the 40-Hz ASSR amplitude and the total GABA level. However, we did not find a formal relationship between age and the GM GABA concentration. Also, neither the amplitude of the 40-Hz ASSR in quiet nor the GM GABA concentration was significantly related to auditory performance. However, the relationship between the GABA concentration and gamma synchrony was specific to the left hemisphere in the older age group, indicating it may be related to differences in auditory cortex asymmetry between young and older adults. We previously showed an aging-related reduction in the asymmetry of auditory cortical GM GABA concentrations and GABA/Cr ratios (Dobri & Ross, 2021). Compared to young adults, older adults show a substantial increase in the right-ear advantage, where listeners are better able to perceive speech stimuli presented to the right ear compared to the left when simultaneously presented with different words to each ear (Jerger et al., 1994; Kimura, 1967; Roup, 2011). Because input to the right ear is predominantly routed to the contralateral left auditory cortex, this suggests increasing dominance of the left hemisphere for auditory processing. In this case, we expect older adults to prefer the left hemisphere in controlling synchrony in the binding network. For the binaural stimulus used in our study, 40-Hz gamma synchrony may not depend as strongly on local GABA levels in young adults because balanced bilateral interactions generate synchrony.

In addition to the GM GABA concentration in brain matter, we investigated how the total amount of auditory cortical GABA related to 40-Hz gamma oscillations. We previously reported that an aging-related decrease in the total GABA level in the right auditory cortex partially mediated the relationship between age and poorer SIN performance in older adults. We hypothesized this was because decreasing GABA levels affected gamma synchrony (Dobri & Ross, 2021). In the current study, we found an aging-related increase in 40-Hz synchrony in the sensory network was associated with poorer SIN performance. Thus, we expected lower total GABA levels to be associated with a larger amplitude of 40-Hz oscillations in the sensory network. Although we found only a tendency toward this relationship when testing it directly, our finding that total GABA levels mediated the relationship between gamma amplitude and SIN loss supports this hypothesis. In addition to controlling gamma synchrony, auditory cortex GABA plays a crucial role in the response selectivity of auditory cortical neurons (Foeller et al., 2001; Fuzessery & Hall, 1996; Kaur et al., 2004; J. Wang et al., 2002). Also, the total GABA level was related to neural distinctiveness, the degree to which activation patterns differ for different auditory stimuli (Lalwani et al., 2019). This suggests tha total GABA levels may be more relevant for controlling the sensory response to more complex auditory stimuli than the simple AM tone used in our study.

The GABA-gamma relationship we found has implications beyond aging. Alterations in 40-Hz ASSRs in schizophrenia have been discussed as indicating an imbalance between excitatory and inhibitory mechanisms (Larsen, 2022; Sivarao et al., 2016). Recent evidence for this comes from a study of individuals with 22q11.2 deletion syndrome, a genetic disorder which affects the GABAergic system. Carriers of the deletion are at high risk of developing psychiatric disorders and showed decreased synchronization of 40-Hz ASSRs (Mancini et al., 2022). Our results indirectly support the hypothesis that deficits in GABAergic neurotransmission are related to altered 40-Hz ASSRs in disorders such as schizophrenia.

## 5 Conclusion

Our MEG and MRS study investigated the neural mechanisms underlying central mechanisms of speech-in-noise loss in aging. Increasing 40-Hz gamma synchrony in aging may result in greater interference between speech and noise sounds, making it more difficult for the aging brain to bind them into distinct perceptual objects. The correlation between the auditory cortical GABA concentration in gray matter and 40-Hz gamma synchrony reflected the role of GABA in the internal generation of gamma oscillations.

## Data Availability

The institutional policy is that a formal data-sharing agreement is required to share data with outside researchers. Therefore, all data and code used in this study will be made available upon reasonable request made to the corresponding author, Simon Dobri (simon.dobri@mail.utoronto.ca).
